# Abnormal low expression of SFTPC promotes the proliferation of lung adenocarcinoma by enhancing PI3K/AKT/mTOR signaling transduction

**DOI:** 10.18632/aging.205191

**Published:** 2023-11-12

**Authors:** Baile Zuo, Lin Wang, Xiaoyan Li, Xin Li, Jinping Wang, Yanlu Xiong, Jie Lei, Xi Zhang, Yifan Chen, Qiongwen Liu, Jinke Jiao, Mengru Sui, Jinhan Fan, Ningxue Wu, Zewen Song, Guoyin Li

**Affiliations:** 1 Henan Key Laboratory of Immunology and Targeted Drugs, School of Medical Technology, Xinxiang Medical University, Xinxiang, Henan, China; 2 Department of Geriatrics, Xijing Hospital, The Air Force Military Medical University, Xi’an, Shaanxi, China; 3 Department of Blood Transfusion, Shanxi Provincial People’s Hospital, Affiliate of Shanxi Medical University, Taiyuan, Shanxi, China; 4 Department of Geriatric Medicine, Shanxi Provincial People’s Hospital, Affiliate of Shanxi Medical University, Taiyuan, Shanxi, China; 5 Department of Ultrasound, Shanxi Provincial People’s Hospital, Affiliate of Shanxi Medical University, Taiyuan, Shanxi, China; 6 Department of Thoracic Surgery, Tangdu Hospital, Fourth Military Medical University, Xi’an, Shaanxi, China; 7 Department of Oncology, The Third Xiangya Hospital of Central South University, Changsha, Hunan, China; 8 College of Management, Zhejiang Shuren University, Hangzhou, Zhejiang, China; 9 College of Life Science and Agronomy, Zhoukou Normal University, Zhoukou, Henan, China; 10 MOE Key Laboratory of Modern Teaching Technology, Center for Teacher Professional Ability Development, Shaanxi Normal University, Xi’an, Shaanxi, China; 11 Academy of Medical Science, Zhengzhou University, Zhengzhou, Henan, China

**Keywords:** SFTPC, lung adenocarcinoma, prognosis, PI3K/AKT/mTOR signaling, proliferation

## Abstract

The abnormality of surfactant protein C (SFTPC) has been linked to the development of a number of interstitial lung diseases, according to mounting evidence. Nonetheless, the function and mechanism of SFTPC in the biological progression of lung adenocarcinoma (LUAD) remain unclear. Analysis of public datasets and testing of clinical samples suggested that SFTPC expression was abnormally low in LUAD, which was associated with the onset and poor prognosis of LUAD. The SFTPC-related risk score was derived using least absolute shrinkage and selection operator Cox regression as well as multivariate Cox regression. The risk score was highly correlated with tumor purity and tumor mutation burden, and it could serve as an independent prognostic indicator for LUAD. Low-risk LUAD patients may benefit more from CTLA-4 or/and PD-1 inhibitors. Overall, the risk score is useful for LUAD patient prognostication and treatment guidance. Moreover, *in vitro* and *in vivo* experiments demonstrated that SFTPC inhibits the proliferation of LUAD by inhibiting PI3K/AKT/mTOR signaling transduction. These results reveal the molecular mechanism by which SFTPC inhibits the proliferation of LUAD and suggest that SFTPC could be a new therapeutic target for LUAD.

## INTRODUCTION

Lung cancer is considered as a leading cause of cancer-related mortality worldwide in 2020, accounting for 18.4% [1]. Small-cell lung cancer (SCLC) and non-small-cell lung cancer (NSCLC) are the two subtypes of lung cancer. NSCLC accounts for about 85% of all lung cancer cases. Lung adenocarcinoma (LUAD) is the most common histological subtype of NSCLC, and its incidence is rising more rapidly than lung squamous cell carcinoma (LUSC) [2, 3]. As early-stage LUAD is prone to metastasis and two-thirds of patients have advanced disease at diagnosis, their prognosis is poor, with a 5-year survival rate below 20% [4, 5]. Despite diagnostic and therapeutic advances over the past few decades, the outlook for patients with LUAD remains dismal. Therefore, it remains urgent to investigate new therapeutic targets for LUAD and to develop precise prognostic models.

Pulmonary surfactant (PS) is a lipoprotein compound primarily composed of phospholipids, with approximately 10% protein content [6]. The pulmonary surfactant protein family consists of four members: surfactant protein A (SFTPA), surfactant protein B (SFTPB), surfactant protein C (SFTPC), and surfactant protein D (SFTPD), which play important roles in diverse aspects of surfactant structure, function, and metabolism [7]. SFTPA and SFTPD are hydrophilic proteins that regulate the pulmonary immune system, whereas SFTPB and SFTPC are hydrophobic proteins that reduce pulmonary surface tension [8].

SFTPC is only expressed in type II alveolar cells, unlike other members of its family. Not only is SFTPC heavily involved in surfactant protein-derived innate immunity, but it is also linked to the development of a number of interstitial lung diseases [9]. According to studies, SFTPC is abnormally low expressed in LUAD and closely linked to a poor prognosis for patients [10]. *In vivo* and *in vitro*, SFTPC overexpression significantly inhibits the proliferation of NSCLC cells. In addition, the expression levels of matrix metalloproteinases MMP-2 and MMP-9 in alveolar macrophages of SFTPC knockout mice were significantly elevated, which was closely associated with tumor occurrence and development [11]. However, the specific mechanism by which SFTPC inhibits LUAD development has yet to be identified.

We discovered in this study that SFTPC was downregulated in LUAD, which was associated with a poor prognosis. Furthermore, *in vitro* and *in vivo* experiments suggested that SFTPC could inhibit LUAD proliferation by inhibiting the activity of the PI3K/AKT/mTOR signaling pathway. We developed a SFTPC-related risk model, which could serve as an independent prognostic factor for LUAD. We also utilized the SFTPC-related risk model and the clinical characteristics of lung adenocarcinoma patients to develop a nomogram that could accurately assess the prognosis of patients.

## MATERIALS AND METHODS

### Data acquisition and processing

The TCGA LUAD data set’s mRNA expression data and clinical information were retrieved from the Cancer Genome Atlas (TCGA) database (https://portal.gdc.cancer.gov). The GSE31210, GSE10072, GSE43458, GSE32863, GSE46539, GSE72094, GSE41271, and GSE3141 were obtained from the Gene Expression Omnibus (GEO) database (https://ncbi.nlm.nih.gov/gds). All datasets were processed according to the methods outlined in our previous study [12, 13].

### Enrichment analysis

Gene Set Enrichment Analysis (GSEA) was performed on LUAD patients from the TCGA LUAD cohort using the “cluster Profiler” package in R and the “c5.go.bp.v2022.1.Hs.symbols.gmt” gene set database [12].

### Development of SFTPC-related risk score

Based on the level of *SFTPC* expression, patients in the TCGA_LUAD and GSE72094 cohorts were divided into high- and low-expression subgroups, respectively. Then, the “limma” package in R (logFC ≥ 1, FDR ≤ 0.05) was used to identify differentially expressed genes (DEGs). The common genes of DEGs from both datasets were utilized for univariate Cox regression analysis. Incorporating prognosis related genes (*P* < 0.01) into the Least Absolute Shrinkage and Selection Operator (LASSO) regression model generates essential genes and their corresponding coefficients, which was performed by “glmnet” and “survival” packages in R. A new score was calculated for each patient using the following formula: score = ∑i Coefficient (Gene i) * Expression (Gene i). Each patient’s SFTPC-related risk score was calculated using the following formula: risk score = (score-Min) / absolute (Max), which facilitated comparisons between datasets.

### Development and evaluation of the nomogram

The “rsm” package in R was used to develop the nomogram, which was based on the SFTPC-related risk score and clinical characteristics including age, tumor purity, gender, tumor mutation burden (TMB), T stage, M stage, and N stage.

### Cell culture and lentiviral infection

A549 and PC9 human LUAD cancer cells were obtained from the Cell Bank of the Shanghai Institute of Biological Sciences of the Chinese Academy of Sciences. Cells were cultured in a DMEM medium containing 10% FBS under humidified conditions of 5% CO2 and 37° C. Lentivirus was used to infect A549 and PC9 cells to produce stable overexpression or knockdown of *SFTPC*. Sequences of *SFTPC*-RNAi were displayed in Supplementary Table 1.

### CCK-8 assay

A549 and PC9 cells were seeded into 96-well plates at 0, 24, 48, and 72h, respectively. The working solution was prepared according to the instructions (Beyotime C0038, China), and the absorbance value was measured using an enzyme labeling instrument.

### Colony formation assay

A549 and PC9 cells were seeded in 6-well plates (500 cells/well), cultured for 12 days, then fixed with cold methanol, and stained with crystal violet.

### Western blotting

Total proteins were extracted from the cells at the indicated times, and protein concentrations were determined using a BCA kit. The proteins separated on SDS/PAGE gels were transferred to nitrocellulose membranes and immunoblotted with the antibodies listed in Supplementary Table 2.

### Patients and specimens

Patients in the Thoracic Surgery Department of Tangdu Hospital (Xi’an, China) provided fifty-two pairs of human LUAD tissues and adjacent normal tissues. The Ethics Committee of the Tangdu Hospital authorized the use of clinical specimens (202203-039).

### Immunohistochemistry and immunofluorescence assay

The cancerous and adjacent normal tissues of 52 LUAD patients were fabricated into tissue chips, and the protein levels of target genes were determined using immunohistochemical staining. The immunostaining antibodies are listed in Supplementary Table 2. Slides were scanned and digitalized with a Panoramic MIDI (3DHISTECH, Ltd., Budapest, Hungary) and analyzed with a Panoramic Viewer v. 1.15.3 and Nuclear Quant application for PV v.2.0.0.46136, both manufactured by 3DHISTECH. H-Score = ∑ (pi × i) = (percentage of weak intensity ×1) + (percentage of moderate intensity × 2) + (percentage of strong intensity ×3), where I = intensity of staining and pi = percentage of stained tumor cells [14]. A549 and PC9 cells were seeded into a special dish for confocal laser scanning microscopy, fixed with cold methanol, punched with triton X-100 (0.3%), surrounded with BSA, incubated with antibody, re-stained with DAPI, and detected by confocal laser scanning microscopy.

### Xenograft lung adenocarcinoma model

For the xenograft lung adenocarcinoma models, nude BALB/c female mice aged six weeks were obtained from the Model Animal Research Center of Nanjing University (China). The A549 and PC9 cells were injected subcutaneously into the right inguinal area of nude mice (5 × 10^6^ per mouse), respectively. After seven days of tumor loading (Day 0), tumor volume was measured every three days until mice were euthanized. Tumor volume was calculated using the following formula: Volume = (length × width^2^)/2, where the length and width are the longest and shortest axes. The Animal Research Protocol was approved by the Shanxi Provincial People’s Hospital’s Ethics Committee (2021-191).

### Statistical analysis

In this work, the R (4.1.0) software was used for statistical analysis and drawing images. Comparative statistical analysis of the target genes of two subgroups was done using the Wilcox test. Survival analysis was performed between two subgroups of patients using the log-rank test and the Kaplan-Meier method. Spearman analysis was used to determine the correlation between the two genes. The “ggpubr”, “VennDiagram”, “survival”, “glmnet”, “survminer”, “timeROC”, “rms”, “ggpubr”, “ggExtra”, “pheatmap”, “tidyverse”, “ggplot2”, “reshape2”, “maftools”, “limma”, “regplot”, “pec”, “pRRophetic”, “car”, “ridge”, “preprocessCore”, “genefilter”, and “sva” packages in R were used for visualization. Less than 0.05 p-value was considered statistically significant (*, *P* < 0.05; **, *P* < 0.01; ***, *P* < 0.001).

### Availability of data and materials

The datasets generated during and/or analyzed during the present study are available from the corresponding author on reasonable request.

## RESULTS

### SFTPC downregulation is correlated with the onset and poor prognosis in LUAD

**Figure 1:**
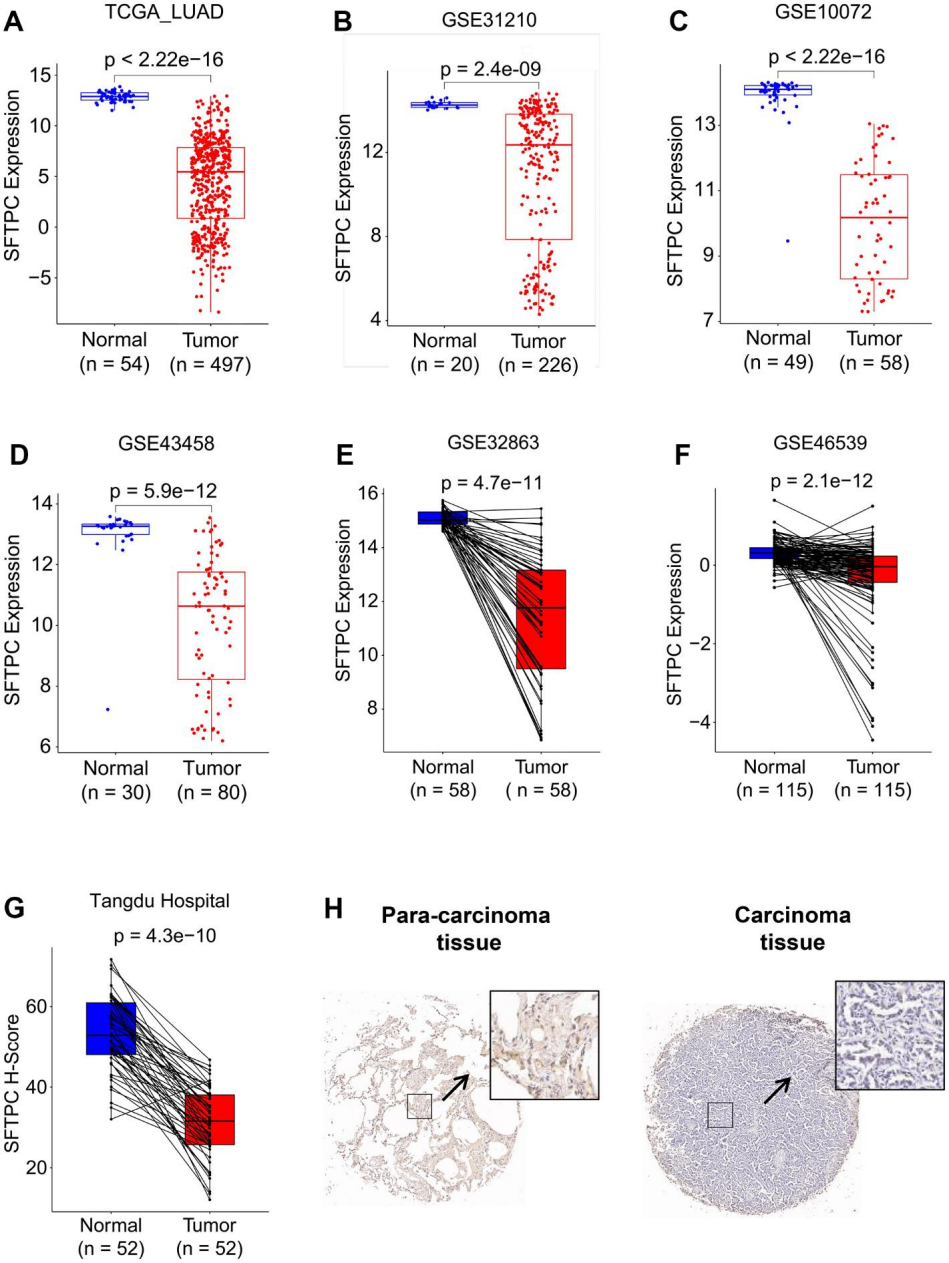
**SFTPC was downregulated in LUAD.** (**A**–**F**) *SFTPC* mRNA levels of LUAD patients in the TCGA_LUAD (**A**), GSE31210 (**B**), GSE10072 (**C**), GSE43458 (**D**), GSE32863 (**E**), and GSE46539 (**F**) cohorts. (**G**, **H**) SFTPC protein levels in LUAD patients in Tangdu Hospital cohort (**G**), and representative IHC staining of SFTPC in carcinoma and para-carcinoma tissues (**H**).

**Figure 2:**
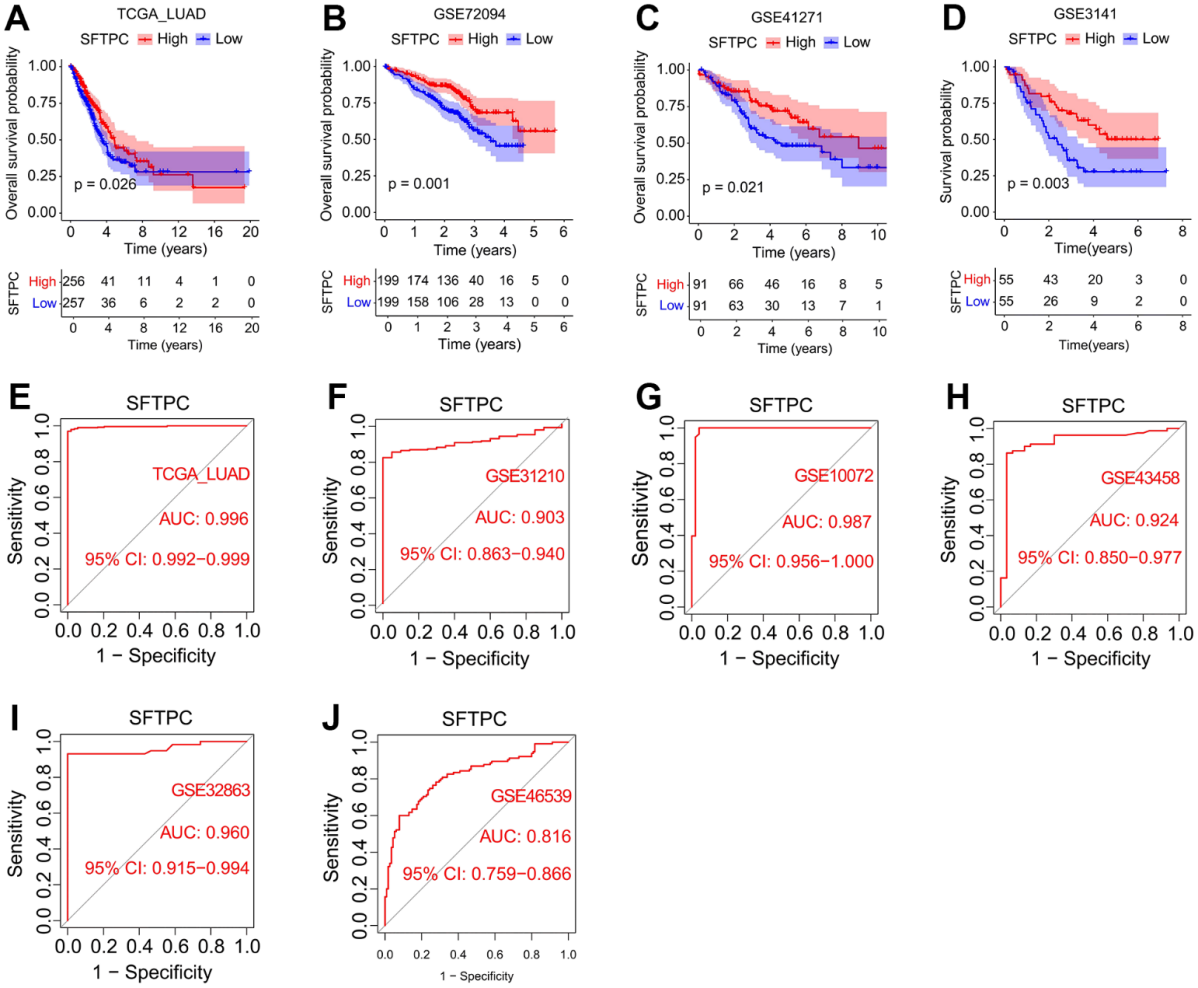
**Correlation analysis between SFTPC and the incidence and prognosis of lung adenocarcinoma.** (**A**–**D**) Kaplan-Meier curves of the OS of LUAD patients in the TCGA_LUAD, GSE72094, GSE41271, and GSE3141 cohorts. (**E**–**J**) SFTPC-dependent ROC analyses of the pathogenic status in TCGA_LUAD (**E**), GSE31210 (**F**), GSE10072 (**G**), GSE43458 (**H**), GSE32863 (**I**), and GSE46539 cohorts.

To investigate the potential role of SFTPC in LUAD, we analyzed the mRNA levels of *SFTPC* in patients from the TCGA LUAD, GSE31210, GSE11072, GSE43458, GSE32863, and GSE46539 cohorts. *SFTPC* was significantly downregulated in carcinoma tissues relative to normal tissues (Figure 1A–1F). Using immunohistochemistry, we then determined the protein level of SFTPC in the carcinoma and para-carcinoma tissues of 52 patients from the Tangdu Hospital. Consistent with the mRNA level, the protein level of SFTPC was significantly decreased in carcinoma tissues (Figure 1G, 1H). The Kaplan-Meier (KM) analysis of patients in the LUAD, GSE72094, GSE41271, and GSE3141 datasets suggested that patients with SFTPC low expression had a shorter overall survival (OS) duration (Figure 2A–2D). Finally, we plotted the ROC curve of SFTPC gene for diagnosing lung adenocarcinoma; the AUC values of SFTPC in the TCGA_LUAD, GSE31210, GSE11072, GSE43458, GSE32863, and GSE46539 cohorts were 0.996, 0.903, 0.987, 0.924, 0.960, and 0.816 respectively (Figure 2E–2J). All of these findings indicated that SFTPC down-regulation in LUAD was significantly associated with disease onset and prognosis.

### Association between immune characteristics and SFTPC

Patients from the TCGA LUAD cohort were divided evenly into two subgroups based on their SFTPC expression levels. To comprehend the effect of SFTPC on the tumor microenvironment (TME), we utilized the ESTIMATE algorithm to analyze the immune and stromal scores of patients from the TCGA LUAD dataset. The findings revealed that patients with low SFTPC expression had lower stromal and immune scores, as well as higher tumor purity (Supplementary Figure 1A, 1B). Subsequently, we analyzed that the expression levels of immune checkpoint genes; *CD27*, *CD274*, *TLA4*, *HAVCR2*, *TIGIT*, and *TOX* were significantly downregulated in SFTPC low expression subgroup (Supplementary Figure 1C). In addition, the infiltration ratios of immune cells such as macrophages M2, T cells CD4 memory resting, mast cells resting, dendritic cells resting, T cells follicular helper, and monocytes were significantly decreased, whereas macrophages M0, T cells CD4 memory activated, and T cells regulatory were significantly increased (Supplementary Figure 1D). Finally, we analyzed the effect of *SFTPC* on 29 functional gene expression signatures and found that *SFTPC* low expression LUAD was characterized by low levels of immune infiltrate and high levels of matrix remodeling, EMT signature, and proliferation rate (Supplementary Figure 1E).

**Figure 3:**
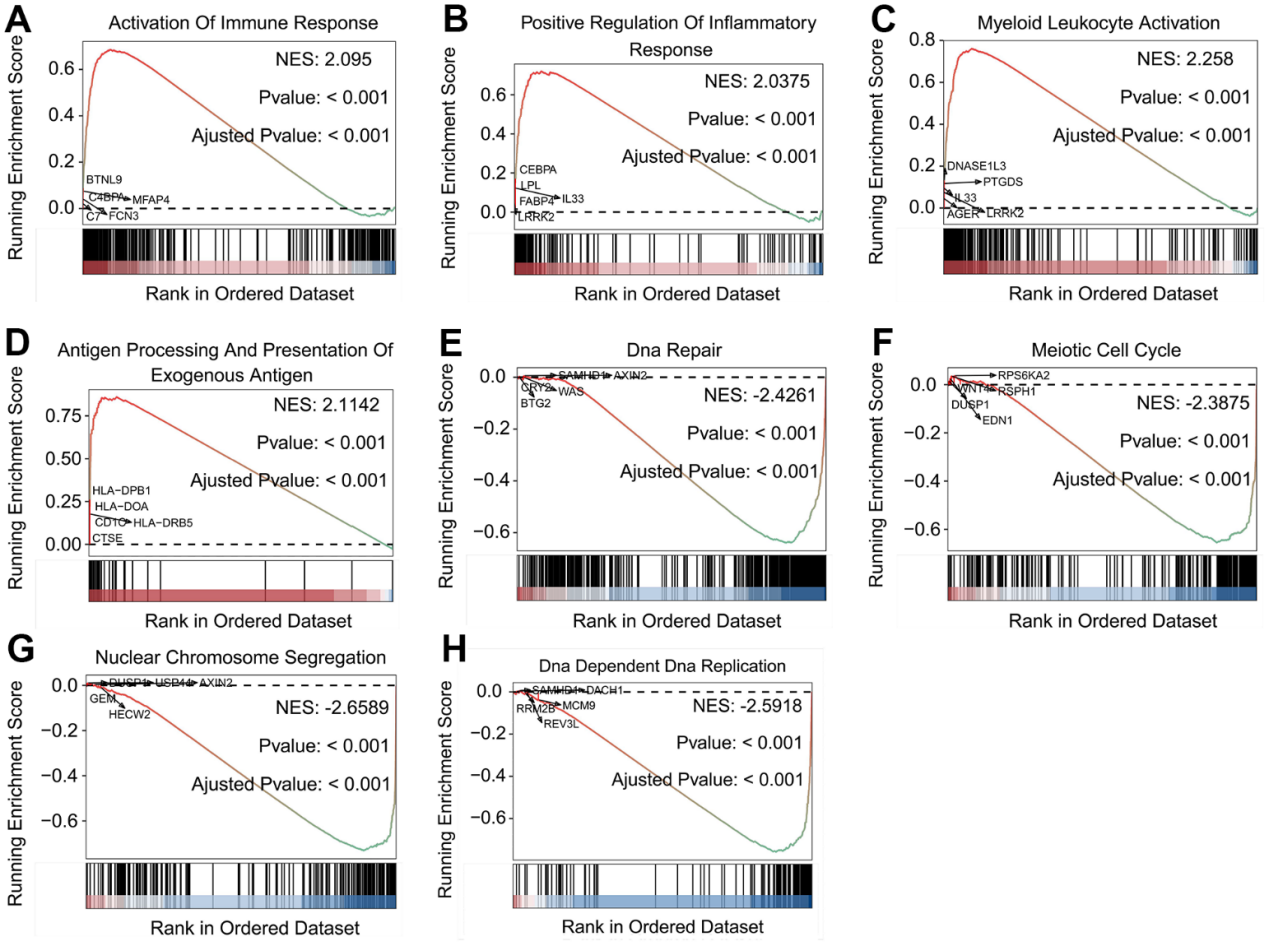
**Biological features of LUAD patients in the stratified SFTPC subgroups.** (**A**–**H**) Examples of GSEA results of LUAD patients with high (**A**–**D**) and low (**E**–**H**) expression of *SFTPC*.

GSEA was performed to investigate the differences between the stratified subgroups of *SFTPC*. LUAD patients with *SFTPC* high expression demonstrated significant enrichment in immune related processes (Supplementary Table 3), including activation of immune response (Figure 3A), positive regulation of inflammatory response (Figure 3B), myeloid leukocyte activation (Figure 3C), and antigen processing and presentation of exogenous antigen (Figure 3D). In addition, the infiltration rate of immune-related cells including macrophages M0 and M2, T cells CD4 memory resting, plasma cell, mast cell resting, and monocytes was significantly reduced (Supplementary Figure 1D). Patients with *SFTPC* low expression were enriched in processes related to cell proliferation, such as DNA repair (Figure 3E), meiotic cell cycle (Figure 3F), nuclear chromosome segregation (Figure 3G), and DNA-dependent DNA replication (Figure 3H). Results indicated that in LUAD, *SFTPC* high expression was associated with tumor immunity, while *SFTPC* low expression was associated with cell proliferation.

### Construction and validation of SFTPC-related risk score

**Figure 4:**
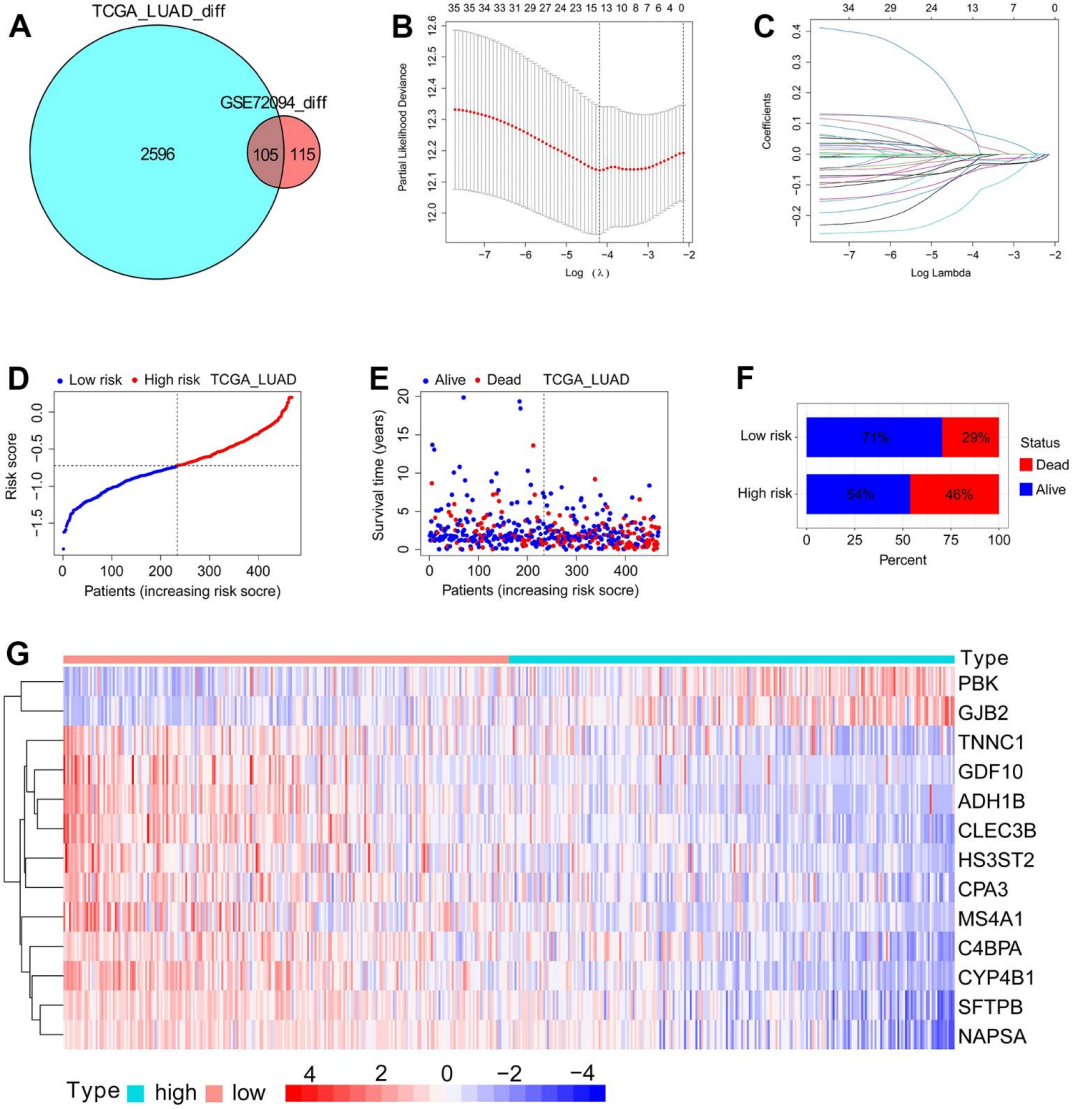
**Development of SFTPC-related risk score using TCGA_LUAD dataset.** (**A**) Venn diagram of DEGs between patients in SFTPC low/high expression subgroups in the TCGA_LUAD and GSE72094 datasets (logFC ≥ 1; adj-*P* < 0.05). (**B**, **C**) LASSO Cox regression model was constructed from 35 DEGs with significant prognostic p-value < 0.01. The 13 essential genes were generated by the optimal profile. (**D**) Distribution and cutoff value of SFTPC-related risk score. (**E**, **F**) OS and survival status of LUAD patients in subgroups with low and high risk. (**G**) Expression heatmap of the 13 essential genes of patients in TCGA_LUAD cohort.

**Figure 5:**
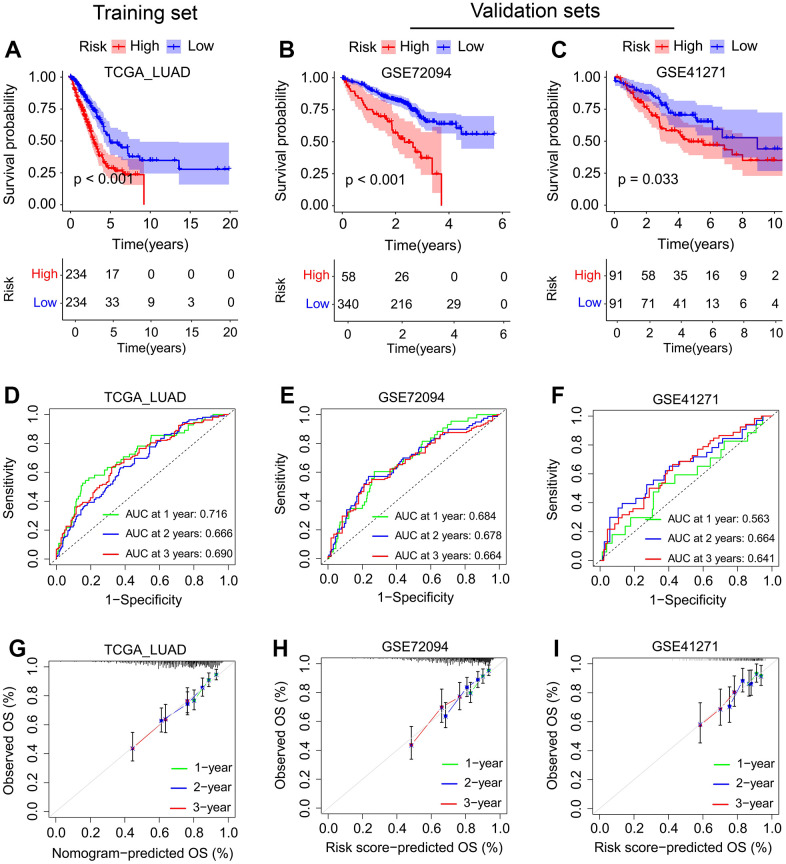
**Evaluation of the SFTPC-related risk score in LUAD.** (**A**–**C**) Kaplan-Meier curves of the OS of LUAD patients in the TCGA_LUAD (**A**), GSE72094 (**B**), and GSE41271 (**C**) cohorts. (**D**–**F**) Time-dependent ROC analyses of risk score regarding the patients’ 1-, 2-, and 3-years survival status in the TCGA_LUAD (**D**), GSE72094 (**E**), and GSE41271 (**F**) cohorts. (**G**–**I**) Calibration curves of SFTPC-related risk score between predicted and observed 1-, 2-, and 3-years survival status in the TCGA_LUAD (**G**), GSE72094 (**H**), and GSE41271 (**I**) cohorts.

To develop an SFTPC-related signature that identifies LUAD patients with different prognoses, we analyzed the DEGs between SFTPC low/high expression subgroups. Figure 4A demonstrates that a total of 2701 and 220 DEGs were identified in the TCGA LUAD and GSE72094 datasets, respectively (logFC ≥ 1; adj-*P* < 0.05). The 105 DEGs from both cohorts were subsequently subjected to univariate Cox analyses in the TCGA LUAD cohort, and 35 of them were significantly associated with the prognosis of patients (*P* < 0.01). Finally, the 35 DEGs were inserted into a LASSO Cox regression model in TCGA_LUAD as described in our previous works [12, 15, 16], and 13 key genes and their corresponding coefficients were obtained (Figure 4B, 4C, 4G). Using the following formula, the SFTPC-related risk score of each patient in a cohort was determined: score = 0.1245*ADH1B - 0.0637*CLEC3B - 0.0417*CYP4B1 - 0.0533*GDF10 - 0.0052*C4BPA - 0.0213*SFTPB - 0.0176*TNNC1 - 0.0153*NAPSA - 0.0647*HS3ST2 - 0.0419*CPA3 - 0.0609*PBK - 0.0805*GJB2 - 0.1682*MS4A1. The TCGA_LUAD cohort served as the training set, with patients assigned equally to the low-risk and high-risk subgroups; patients in the low-risk group have a higher survival rate (Figure 2D–2F). As validation datasets, the GSE72094 and GSE41271 cohorts were used, and based on the risk score, patients in both cohorts were divided into low-risk and high-risk subgroups, respectively. Patients in the low-risk groups had a significantly longer OS in both the training and validation sets compared to those in the high-risk groups (Figure 4A–4C). The area under curves (AUC) values of SFTPC-related risk score in the TCGA_LUAD dataset were 0.716 for one year, 0.666 for two years, and 0.69 for three years; 0.684 for one year, 0.678 for two years, and 0.664 for three years in the GSE72094 dataset; were 0.563 for one year, 0.664 for two years, and 0.641 for three years in the GSE41271 dataset (Figure 5D–5F). Figure 5G–5I displays the 1-, 2-, and 3-year calibration curves of the SFTPC-related risk score for the TCGA LUAD, GSE72094, and GSE41271 cohorts. All of the aforementioned findings suggested that the SFTPC-related risk score could accurately predict the prognosis of LUAD patients.

### Tumor microenvironment landscape of SFTPC-based classification

**Figure 6:**
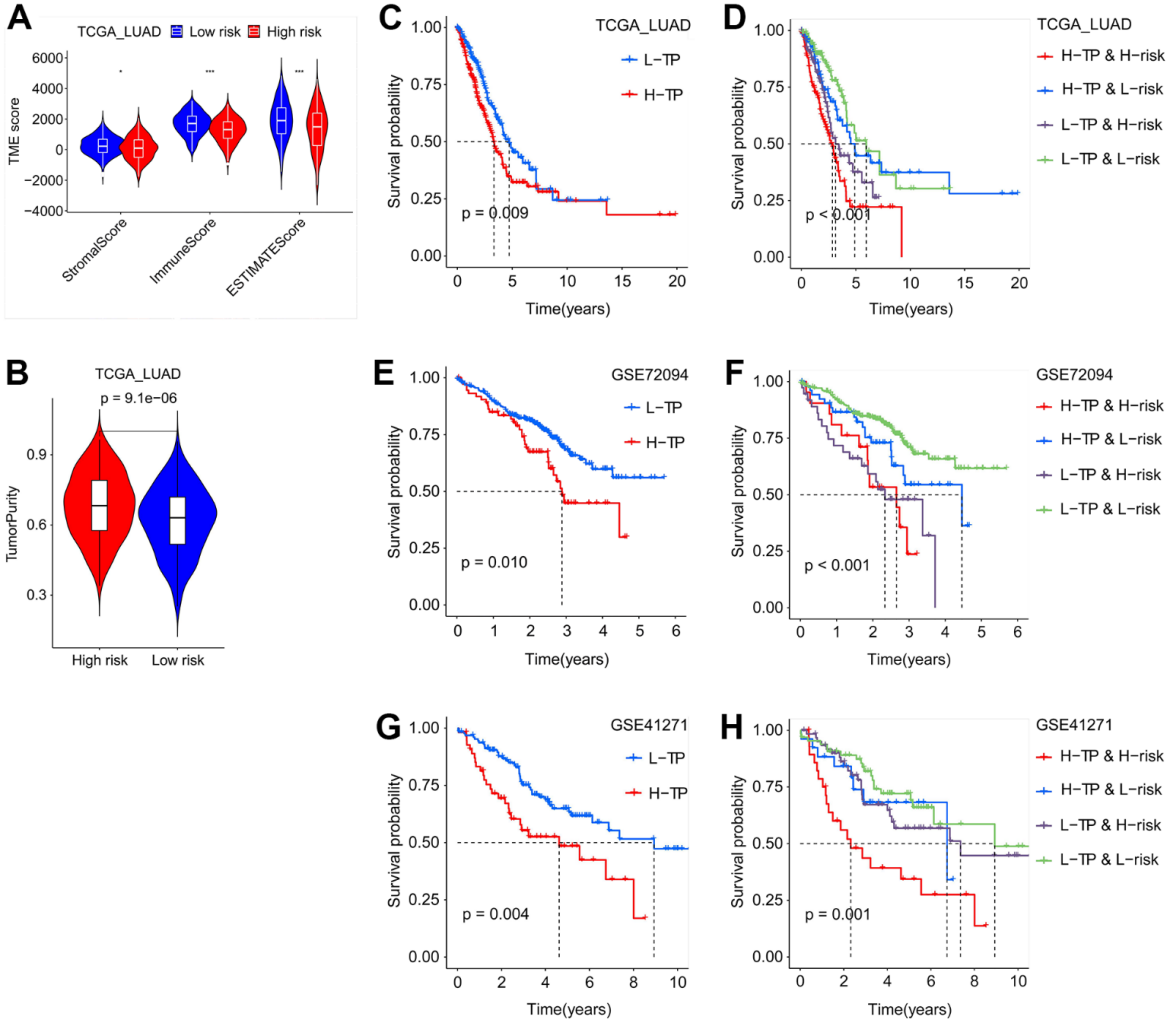
**Tumor purity combined with SFTPC-related risk score to evaluate the prognosis of LUAD patients.** (**A**) The immune and stromal scores of LUAD patients in the TCGA_LUAD cohort, grouping based on the risk score. (**B**) The tumor purity of LUAD patients in the TCGA_LUAD cohort, grouping based on the risk score. (**C**–**H**) Kaplan-Meier curves of the OS of LUAD patients in different subgroups from TCGA_LUAD (**C**, **D**), GSE72094 (**E**, **F**), and GSE41271 (**G**, **H**) cohorts.

Lung adenocarcinoma’s biological progression and prognosis are correlated with the tumor microenvironment (TME) [17, 18]. Patients from the TCGA LUAD, GSE72094, and GSE41271 were divided into low-risk (L-risk) and high-risk (H-risk) subgroups in order to examine the influence of SFTPC-related risk score on TME. In the H-risk groups of the three cohorts, the infiltration ratios of immune cells such as aDCs, DCs, iDCs, mast cells, and neutrophils decreased significantly (Supplementary Figure 2A, 2C, 2E). Moreover, in H-risk groups, the functions of HLC and type II IFN response were also downregulated, whereas the function of MHC class I was upregulated (Supplementary Figure 2B, 2D, 2F). In the TCGA LUAD dataset, we discovered that the infiltration ratios of immune cells including T cells memory resting/activated, monocytes, mast cells resting, macrophages M0/M1, dendritic cells resting, and B cells memory were significantly associated with the expression of 13 essential genes (Supplementary Figure 3). Additionally, we observed that patients in the H-risk group have lower stromal and immune scores, as well as higher tumor purity (Figure 6A, 6B).

To investigate the effect of tumor purity on the prognosis of LUAD, patients in the TCGA_LUAD, GSE72094, and GSE41271 cohorts were divided equally into low tumor purity (L-TP) and high tumor purity (H-TP) subgroups respectively. In the three aforementioned datasets, KM analysis indicated that L-TP patients had a longer OS (Figure 6C, 6E, 6G). In addition, we conducted a comprehensive analysis of the effect of SFTPC-related risk score and tumor purity on patient prognosis and discovered that, across the three datasets, patients in the L-TP and L-risk group had the best prognosis (Figure 6D, 6F, 6H). All of these findings suggested that the SFTPC-related risk score was highly correlated with tumor purity and patient prognosis.

### TMB of SFTPC-related classification

**Figure 7:**
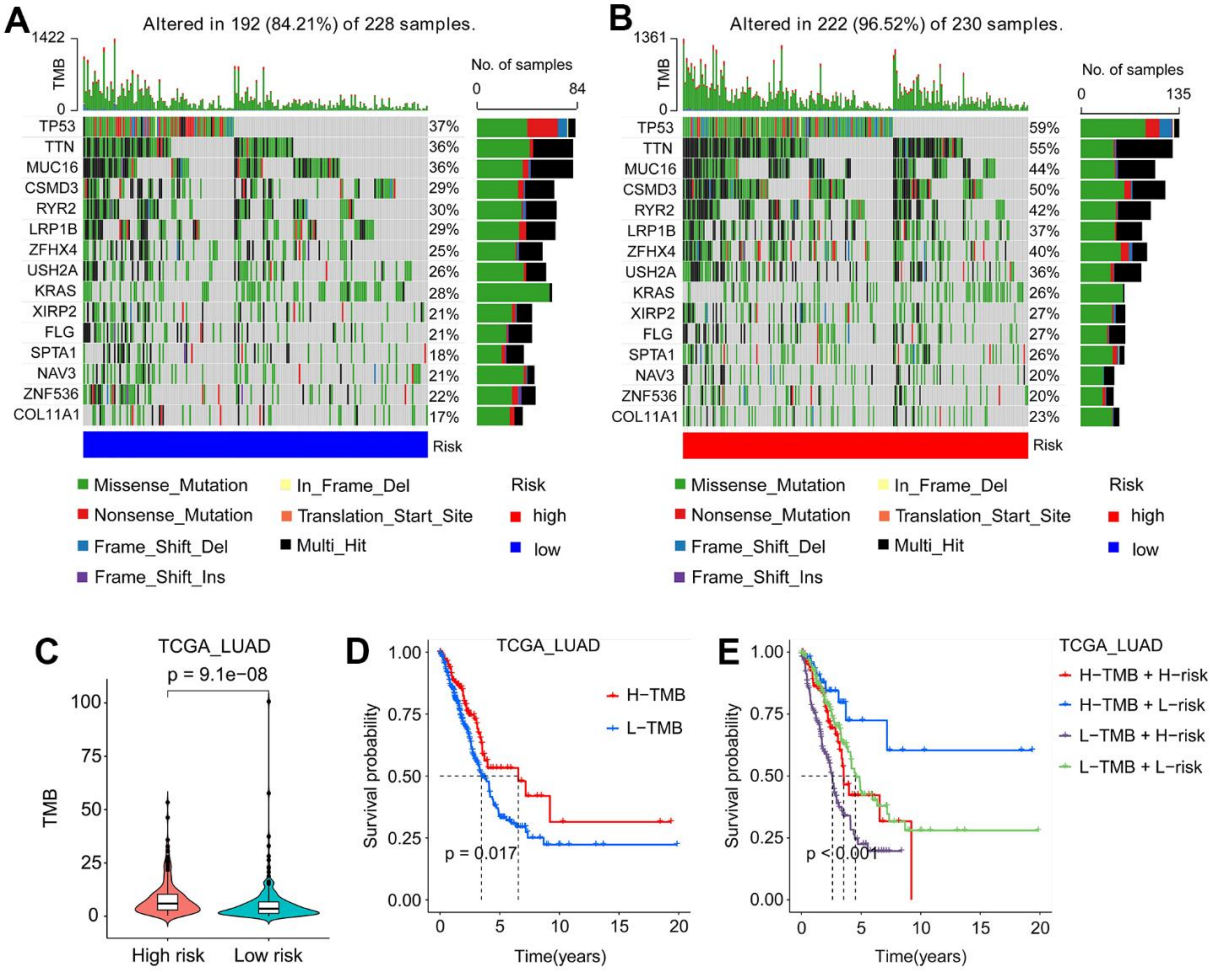
**Mutation signatures of LUAD patients.** (**A**, **B**) Waterfall plots of mutation genes in patients from the TCGA_LUAD cohort, low-risk (**A**) and high-risk (**B**) subgroups. (**C**) In the TCGA_LUAD cohort, patients in the high-risk subgroup had a higher TMB. (**D**) Kaplan-Meier curves of the OS of LUAD patients in the L-TMB and H-TMB subgroups from the TCGA_LUAD cohort. (**E**) Kaplan-Meier curves of the OS of LUAD patients in the H-TMB and H-risk, H-TMB and L-risk, L-TMB and H-risk, and L-TMB and L-risk subgroups from TCGA_LUAD cohort. L-TMB: low tumor mutation burden; H-TMB: high tumor mutation burden; L-risk: low-risk; H-risk: high-risk.

Mounting evidence suggested that TMB was an essential biomarker for LUAD prognosis [19, 20]. To investigate the effect of SFTPC-related risk score on TMB, we subdivided TCGA LUAD patients into low-risk (L-risk) and high-risk (H-risk) subgroups. As depicted in Figure 7A, 7B, patients in the L-risk and H-risk subgroups displayed distinct mutation characteristics, with the L-risk group exhibiting a lower TMB (Figure 7C). In the L-risk group, the top five genes with the highest mutant frequency were TP53 (37%), TTN (36%), MUC36 (39%), RYR2 (30%), and CSMD3 (29%); in the H-risk group, the top five genes with the highest mutant frequency were TP53 (59%), TTN (59%), CSMD3 (50%), MUC16 (44%) and RYR2 (42%) (Figure 7A, 7B). Results of KM analysis indicated that patients in the H-TMB group had a longer OS (Figure 7D). In addition, we analyzed the influence of SFTPC-related risk score and TMN on LUAD prognosis and found that patients in the H-TMB and L-risk subgroup had the most favorable prognosis (Figure 7E).

### Guidance of SFTPC-related risk score in LUAD therapy

**Figure 8:**
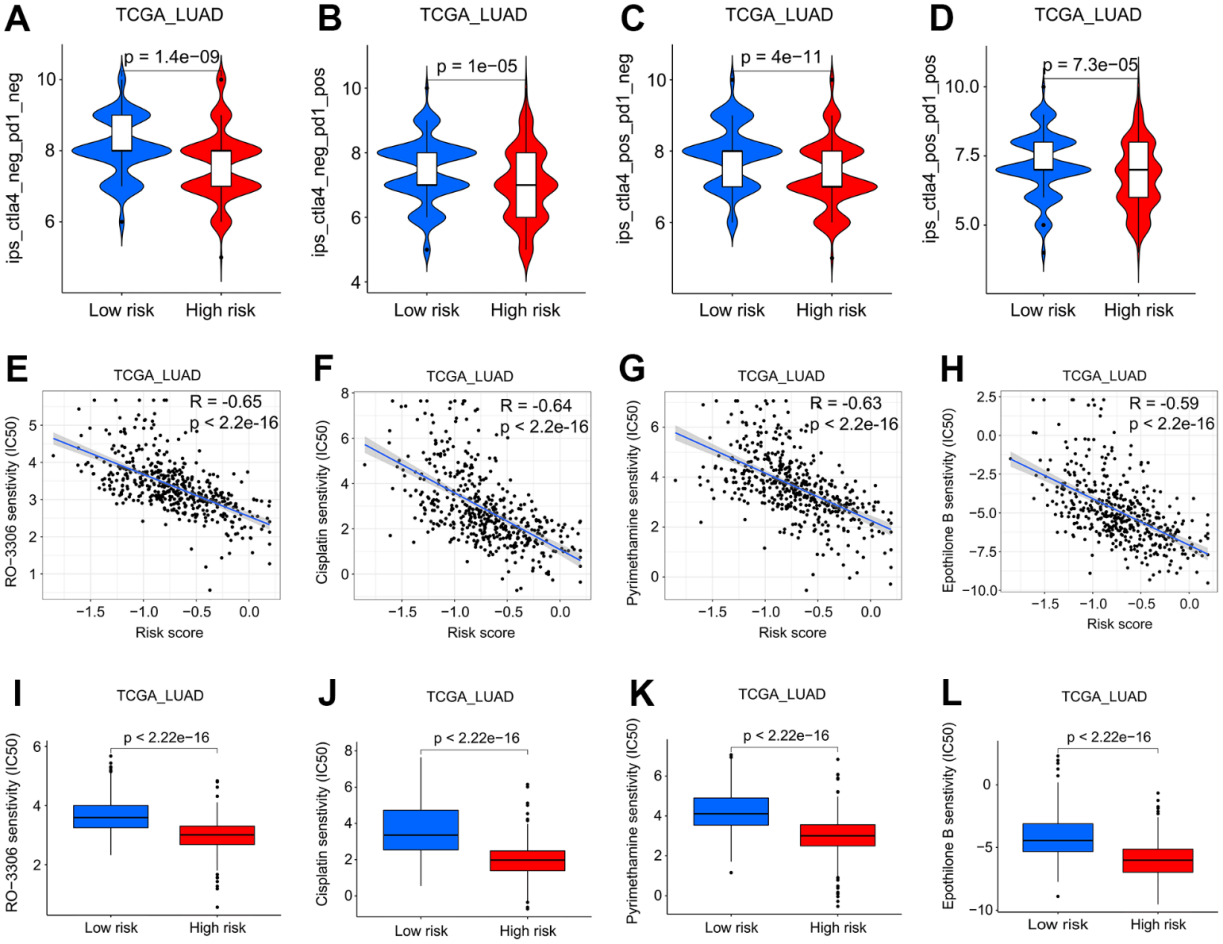
**Screening of potential drugs for LUAD patients.** (**A**–**D**) Patients at low risk will benefit more from CTLA4 and/or PD1 antibodies in the TCGA LUAD cohort. (**E**–**H**) Analysis of the correlation between sensitivity of patients to RO-3306, cisplatin, pyrimethamine, and epothilone B and risk score in the TCGA LUAD cohort. (**I**–**L**) Patients in the low-risk subgroup of the TCGA LUAD cohort were more sensitive to RO-3306, cisplatin, pyrimethamine, and epothilone B.

Evidence suggests that immune checkpoint blockade immunotherapies targeting programmed cell death 1 (PD-1) or cytotoxic T-lymphocyte associated protein 4 (CTLA-4) are emerging as a new treatment option for lung cancer patients [21–23]. However, patient response rates to PD-1 and CTLA-4 inhibitors vary widely [24]. How to effectively evaluate the patient’s response to immunosuppressants is a pressing issue in LUAD clinical treatment. We retrieved the clinical data of LUAD patients treated with CTLA-4 or/and PD-1 from The Cancer Immunome Atlas (TCIA) database and observed that high-risk patients may benefit more from immunotherapy (Figure 8A–8D). We then conducted a correlation analysis between the SFTPC-related risk score and the sensitivity of 165 drugs. The risk score was negatively correlated with RO-3306, cisplatin, pyrimethamine, and epothilone B et al. (R ≤ -0.59; p < 0.001), and patients in the low-risk group could benefit more from 158 drugs, while those in the high-risk group were more sensitive to 7 drugs (Supplementary Table 4, Figure 8E–8L). The aforementioned findings suggested that the SFTPC-related risk score could guide LUAD treatment.

### Establishment of a nomogram based on SFTPC-related risk score and clinical characters

**Figure 9:**
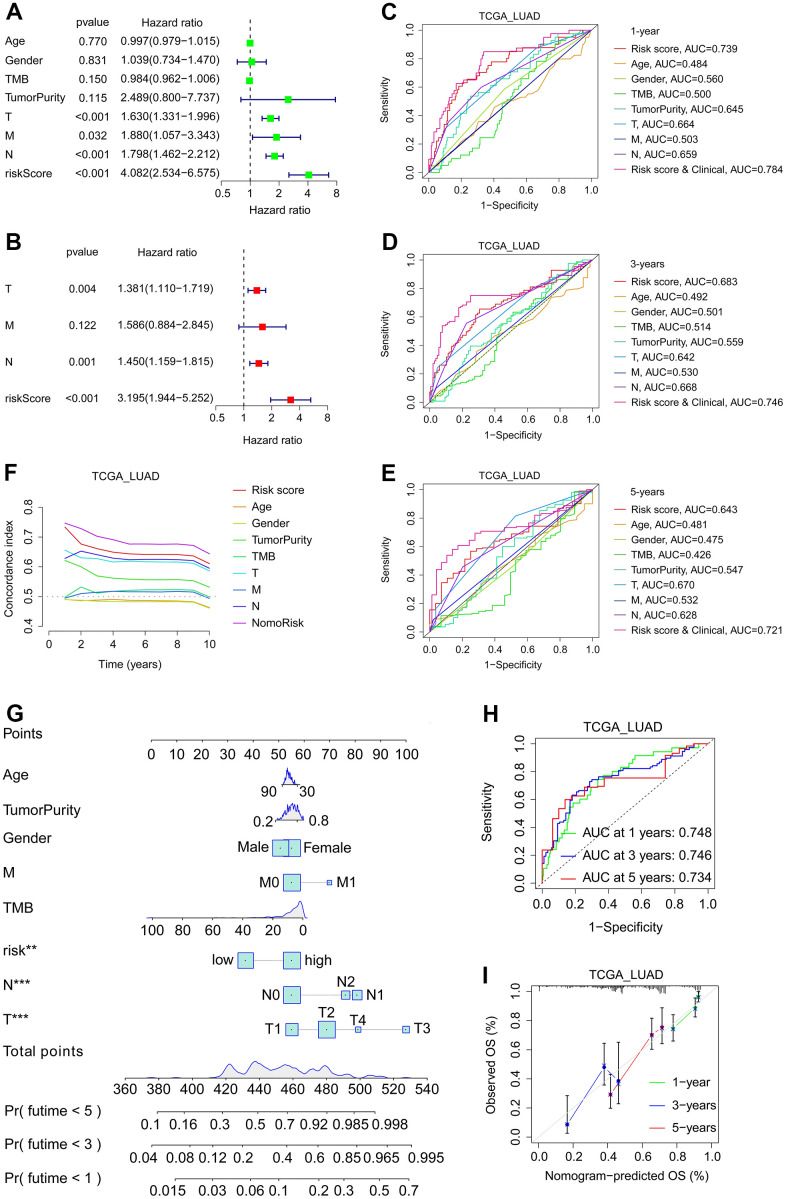
**Development and verification of nomogram in LUAD.** (**A**, **B**) Univariate (**A**) and multivariate (**B**) regression analysis related to OS of patients in TCGA_LUAD cohort. (**C**–**E**) In the TCGA LUAD cohort, time-dependent ROC analyses of the patients’ 1- (**C**), 3- (**D**), and 5-year (**E**) survival status based on their risk score and/or clinical characteristics. (**F**) The C-index curves of risk score and clinical features. (**G**) The nomogram is based on gender, age, tumor purity, T stage, N stage, M stage, TMB, and risk. (**H**) Time-dependent ROC analyses of the patients’ 1-, 3-, and 5-year survival status based on the nomogram. (**I**) The calibration curves of the nomogram between predicted and observed 1-, 3- and 5-year OS in the TCGA_LUAD cohort.

A nomogram was developed using SFTPC-related risk score and clinical characteristics to assess patient prognosis. Initially, univariate and multivariate regression analyses were performed on the TCGA_LUAD cohort, and T stage, M stage, and risk score were identified as independent prognostic factors for LUAD (Figure 9A, 9B). Subsequently, we plotted Receiver Operating Characteristic Curve (ROC) curves for one, three, and five years and discovered that the risk score and clinical group always had the greatest area under the curve (AUC) value (Figure 9C–9E). Then, using age, tumor purity, gender, TMB, T stage, M stage, N stage, and risk, we drew the nomogram (Figure 9G). The C-index curves revealed that nomorisk had the greatest value, indicating that it had the maximum prognostic accuracy for LUAD prognosis (Figure 9F). The AUC values of the nomogram in the TCGA_LUAD dataset were 0.748 for one year, 0.746 for two years, and 0.734 for three years, and the calibration curves of patients at one, three, and five years confirmed the accuracy of the nomogram (Figure 9H, 9I). These findings confirmed that the nomogram was a superior model for LUAD prognosis prediction than individual risk factors.

### SFTPC inhibited the proliferation of LUAD *in vitro* and *in vivo*

**Figure 10:**
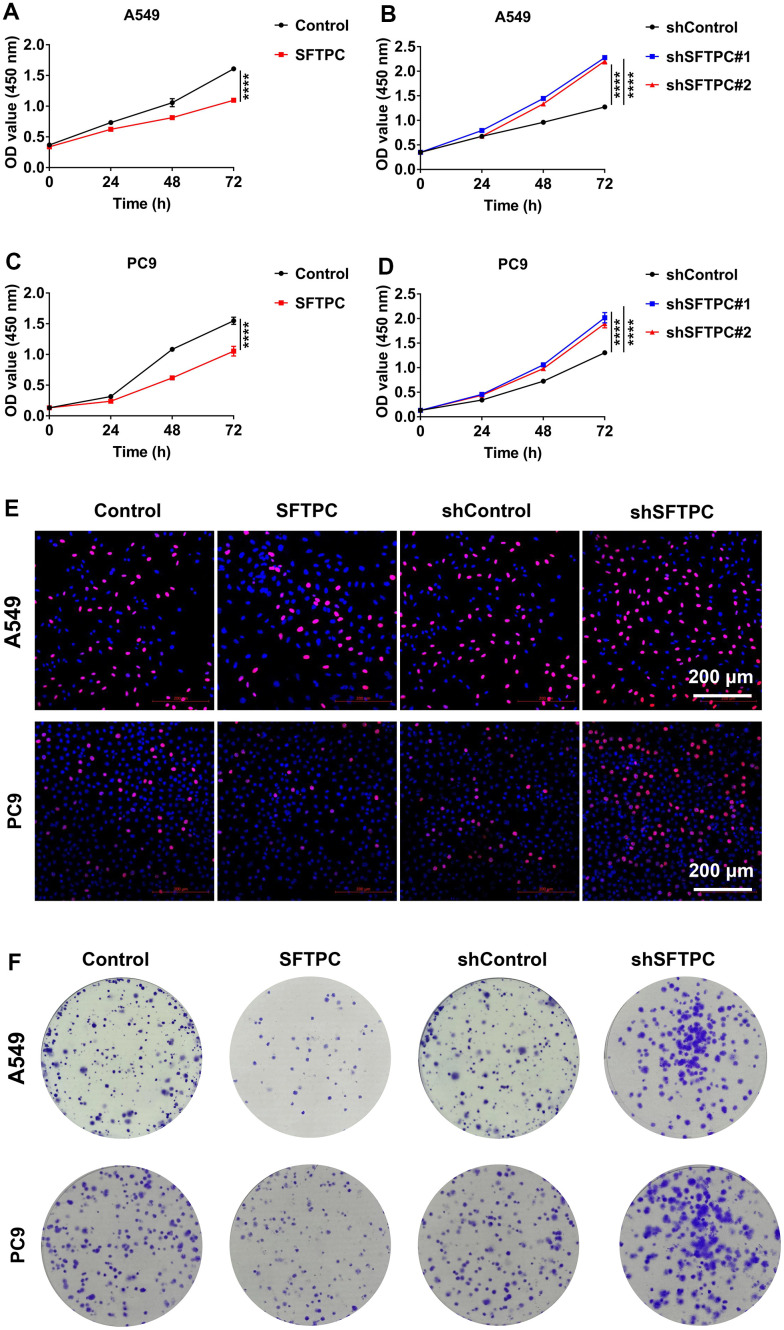
**SFTPC can inhibit the proliferation of LUAD *in vitro*.** (**A**–**D**) CCK-8 assay for A549 and PC9 cells stably overexpressing (**A**, **C**) or inhibiting *SFTPC* expression (**B**, **D**). (**E**) Immunofluorescence analysis of A549 and PC9 cells stably overexpressing or knocking down *SFTPC*. (**F**) Colony formation assays for A549 and PC9 cells stably overexpressing or knocking down *SFTPC*. *, *P* < 0.05; **, *P* < 0.01; ***, *P* < 0.001.

**Figure 11:**
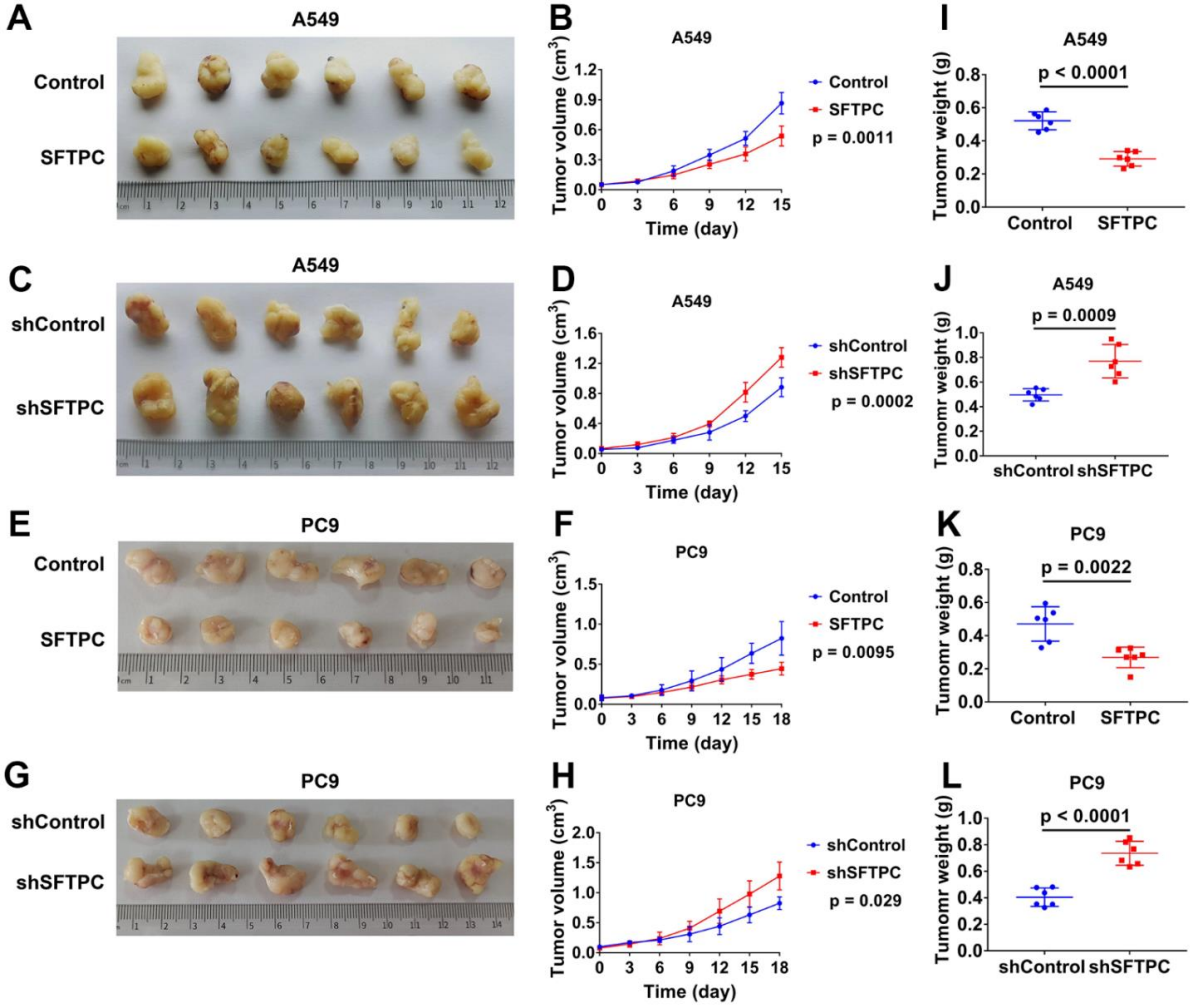
**SFTPC can inhibit the proliferation of LUAD *in vivo*.** (**A**–**D**) Xenograft models and the tumor growth curves of LUAD tumor derived from A549 cells: overexpression of *SFTPC* significantly inhibited the proliferation of A549 cells *in vivo* (**A**, **B**), while knockdown of *SFTPC* significantly promoted their proliferation (**C**, **D**). (**E**–**H**) Xenograft models and the tumor growth curves of LUAD tumor derived from PC9 cells: overexpression of *SFTPC* significantly inhibited the proliferation of PC9 cells *in vivo* (**E**, **F**), while knockdown of *SFTPC* significantly promoted their proliferation (**G**, **H**). The volume of the tumor was measured every three days until the mice were euthanized, after which the tumor growth curves were drawn. (**I**–**L**) The weights of tumors were significantly decreased in the *SFTPC* overexpression groups (**I**–**K**), whereas they significantly increased in the *SFTPC* knockdown groups (**J**). After euthanizing the mice tumors were collected and weighed.

To investigate the function of SFTPC in LUAD, we used lentivirus to knock down and overexpress *SFTPC* in A549 and PC9 cells respectively. After conducting the CCK-8 assay, we discovered that knocking down *SFTPC* significantly increased the proliferation ability of A549 and PC9 cells, whereas over expressing *SFTPC* significantly decreased their proliferation ability (Figure 10A–10D). Subsequently, we conducted the EdU experiment, which suggested that knocking down *SFTPC* significantly increased the proliferation ability of A549 and PC9 cells, whereas over expressing *SFTPC* dramatically decreased their proliferation ability (Figure 10E). The results of the plate cloning assay indicated that knocking down SFTPC significantly increased the cloning ability of A549 and PC9 cells, whereas overexpressing SFTPC significantly decreased their cloning ability (Figure 10F). These findings demonstrated that SFTPC inhibits the proliferation of LUAD cells *in vitro*. To further confirm the antitumor effect of SFTPC, we generated xenograft LUAD models by injecting A549 and PC9 cells subcutaneously into nude mice. We measured tumor volumes every three days until the mice were euthanized and drew growth curves for the tumors. We observed that knockdown of SFTPC significantly promoted the proliferation of A549 and PC9 cells *in vivo*, while overexpression of SFTPC significantly inhibited their proliferation (Figure 11A–11H). After euthanizing mice, tumors were collected and weighed. Compared to the control group, the weights of tumors in the SFTPC knockdown group were significantly higher, while those in the SFTPC overexpression group were significantly lower (Figure 11I–11L). All of these findings suggested that SFTPC could inhibit the proliferation of LUAD cells *in vitro* and *in vivo*.

### SFTPC inhibits PI3K/AKT/mTOR pathway activity in LUAD

**Figure 12:**
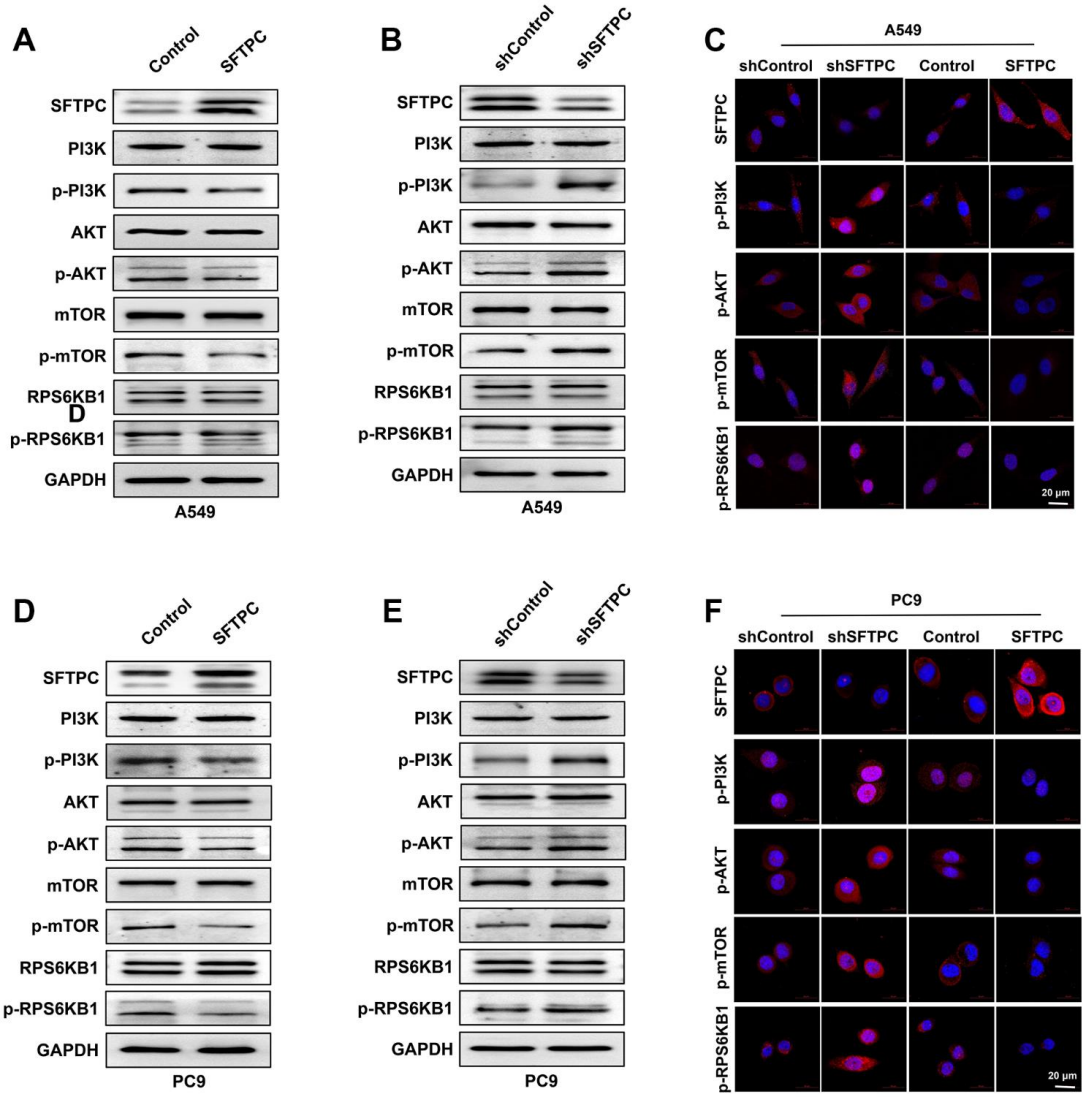
**SFTPC can inhibit PI3K/AKT/mTOR signal transduction.** (**A**–**C**) A549 cells overexpressing or knocking down SFTPC were subjected to Western blot (**A**, **B**) and immunofluorescence (**C**) assay. (**D**–**F**) A549 cells were subjected to Western blot (**D**, **E**) and immunofluorescence (**F**) assays while overexpressing or knocking down *SFTPC*.

To determine the molecular mechanism by which SFTPC inhibits LUAD proliferation, a Western blotting assay was performed initially. Overexpression of *SFTPC* in A549 and PC9 cells significantly reduced the phosphorylation of PI3K, AKT, mTOR, and RPS6KB1 (Figure 12A–12C). Additionally, we observed that knocking down *SFTPC* in A549 and PC9 significantly increased the phosphorylation levels of PI3K, AKT, mTOR, and RPS6KB1 (Figure 12B, 12D). Subsequently, we also conducted immunocytochemical assays on A549 and PC9 cells, and the outcomes were consistent with the WB experiments (Figure 12E, 12F). Using immunohistochemistry, we determined the protein levels of SFTPC, PI3K, p-PI3K, AKT, p-AKT, mTOR, p-mTOR, RPS6KB1, and p-RPS6KB1 in LUAD carcinoma and para-carcinoma tissues. SFTPC protein levels were downregulated in tumor tissues, whereas PI3K, p-PI3K, AKT, p-AKT, mTOR, p-mTOR, RPS6KB1, and p-RPS6KB1 protein levels were upregulated (Figure 1G, 1H and Supplementary Figure 4A–4P). All of the aforementioned results suggested that SFTPC inhibits the PI3K/AKT/mTOR pathway in LUAD.

## DISCUSSION

The *SFTPC* gene is located on the short arm of human chromosome 8 and encodes a transmembrane peptide with a molecular weight of 21 kDa and 197 amino acids [25]. The ProSFTPC protein comprises four domains: a cytosolic domain on the N-terminus, a transmembrane helix, an unstructured linker domain, and a BRICHOS domain on the C-terminus. Prior to secretion, proSFTPC is moved from multivesicles to AT2-specific lamellar bodies. Cleavage of the C- and N-termini sequentially generates mature SFTPC [26]. SFTPC, the smallest surfactant-associated protein, plays a crucial role in stabilizing the surfactant film and recycling surfactant by facilitating lipid movement between sheets and vesicles [27]. Consistent with Zhang et al. [28], this study confirmed that SFTPC was abnormally downregulated in LUAD and was closely associated with a patient’s poor prognosis. However, the molecular mechanisms by which SFTPC is downregulated in LUAD and how it inhibits LUAD’s biological progression remain unknown. This study found that SFTPC was closely associated with LUAD’s TME and TMB (Figures 5A, 5B, 6A–6C).

TME is closely associated with the occurrence, progression, and therapeutic efficacy of tumors [29, 30]. TME [31] is composed of immune and stromal cells. Immune infiltration plays an essential role in LUAD prognosis and therapeutic response [32]. Accumulating evidence suggests that immune checkpoint inhibitors (ICI) are extraordinarily beneficial for NSCLC patients. Nevertheless, according to certain clinical trials, lung cancer patients have a low overall response rate to ICI [33, 34]. High expression of PD-1 and/or CTLA-4 can downregulate T-cell activity, thereby reducing the efficacy of immunotherapy and facilitating the immune evasion of tumor cells [35, 36]. Predicting the efficacy of PD-1 and CTLA-4 antibodies in the clinical treatment of LUAD has become an urgent matter. Our study found that low-risk patients had a lower tumor purity and benefited more from PD-1 and CTLA-4 antibodies, providing a theoretical foundation for the clinical application of ICI (Figures 5A, 5B, 8A–8D). In addition, we discovered that low-risk LUAD patients were more sensitive to RO-3306, cisplatin, pyrimethamine, and epothilone et al., thereby expanding clinical treatment options (Figure 8E–8L). Furthermore, risk score could serve as an independent prognostic factor for LUAD patients, and its C-index was higher than that of clinical features, demonstrating a distinct advantage in predicting the prognosis of patients (Figure 9).

Previous research has demonstrated that TMB can serve as an indicator of immunotherapeutic sensitivity in numerous carcinomas [37]. The greater the TMB, the greater the number of new antigens that T cells can recognize as non-self, making tumors more immunogenic. In LUAD, elevated TMB may augment immune infiltration and enhance the therapeutic efficacy of PD-L1 antibodies [38]. However, this study demonstrated that patients in the low-risk subgroup had a lower TMB, and derived greater benefits from PD-1 and CTLA-4 antibodies, possibly due to the greater proportion of immune cell infiltration in the low-risk subgroup (Figures 6A, 7A–7C, 8A–8D). The C-index curves demonstrated that the prognostic value of TMB for LUAD was inferior to that of risk score, M stage, T stage, and tumor purity (Figure 9F). Our findings indicated that in LUAD, the risk score was not only closely related to TMB but also possessed greater prognostic ability.

To determine the function of SFTPC in LUAD, *in vitro* and *in vivo* experiments were conducted; the results revealed that SFTPC acted as a tumor suppressor (Figures 10, 11). GSEA results indicated that patients with low SFTPC expression were enriched in functions associated with cell proliferation, such as DNA repair, meiotic cell cycle, and DNA replication (Figure 3). Interestingly, these events are closely related to the PI3K/AKT/mTOR pathway, suggesting that SFTPC may affect the proliferation of LUAD cells by regulating the PI3K/AKT/mTOR signaling pathway.

Subsequently, lentivirus was used to overexpress and knockdown SFTPC expression in A549 and PC9 cells, and Western blotting, and immunofluorescence assays confirmed that SFTPC inhibited PI3K/AKT/mTOR pathway activity (Figure 12 and Supplementary Figure 4). Numerous cellular biological processes, such as cell proliferation, metastasis, and metabolism, are dependent on PI3K/Akt/mTOR pathway [39]. Therefore, small molecule inhibitors targeting the PI3K/AKT/mTOR pathway have garnered considerable interest and have been developed and evaluated in preclinical models and clinical trials [39, 40]. However, targeting a single kinase component within PI3K/AKT/mTOR signaling pathway typically results in tumor growth arrest as opposed to apoptosis, which may be caused by abnormal activation of other compensatory pathways. Hence, developing new targets or combining drugs may be effective strategies for enhancing the anti-tumor effect. Cumulatively, SFTPC is not only closely associated with the onset and prognosis of LUAD, but also has the ability to inhibit the PI3K/AKT/mTOR pathway, making it a potential therapeutic target for LUAD.

## Supplementary Material

Supplementary Figures

Supplementary Tables 1 and 2

Supplementary Table 3

Supplementary Table 4
