# Supplementary material for: Abnormal low expression of SFTPC promotes the proliferation of lung adenocarcinoma by enhancing PI3K/AKT/mTOR signaling transduction
Source: Aging (Albany NY). 2023 Nov 12;15(21):12451–75. doi: 10.18632/aging.205191 (PMC10683597; doi:10.18632/aging.205191)
Supplement: Supplementary Figures [file aging-15-205191-s001.pdf]

SUPPLEMENTARY FIGURES

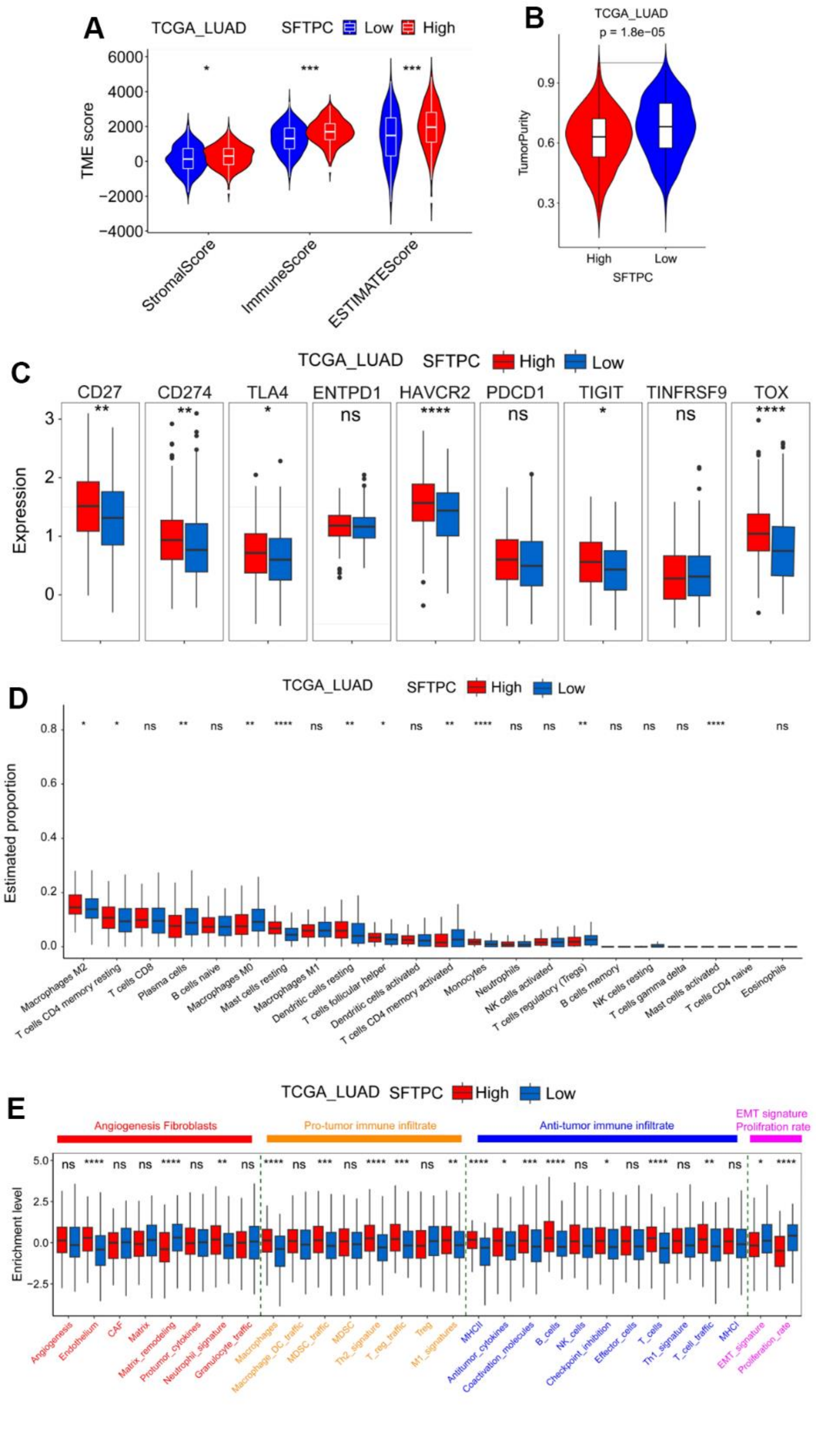

Supplementary Figure 1. Tumor microenvironment landscape of SFTPC-based classification. (**A**) The immune and stromal scores based on SFTPC classification in the TCGA_LUAD cohort. (**B**) The tumor purity based on SFTPC classification in the TCGA_LUAD cohort. (**C**) Expression of immune checkpoint genes based on SFTPC classification in the TCGA_LUAD cohort. (**D**) Distribution of infiltrating immune cells based on SFTPC classification in the TCGA_LUAD cohort. (**E**) Heatmap showed the 29 Fges based on SFTPC classification in the TCGA_LUAD cohort.

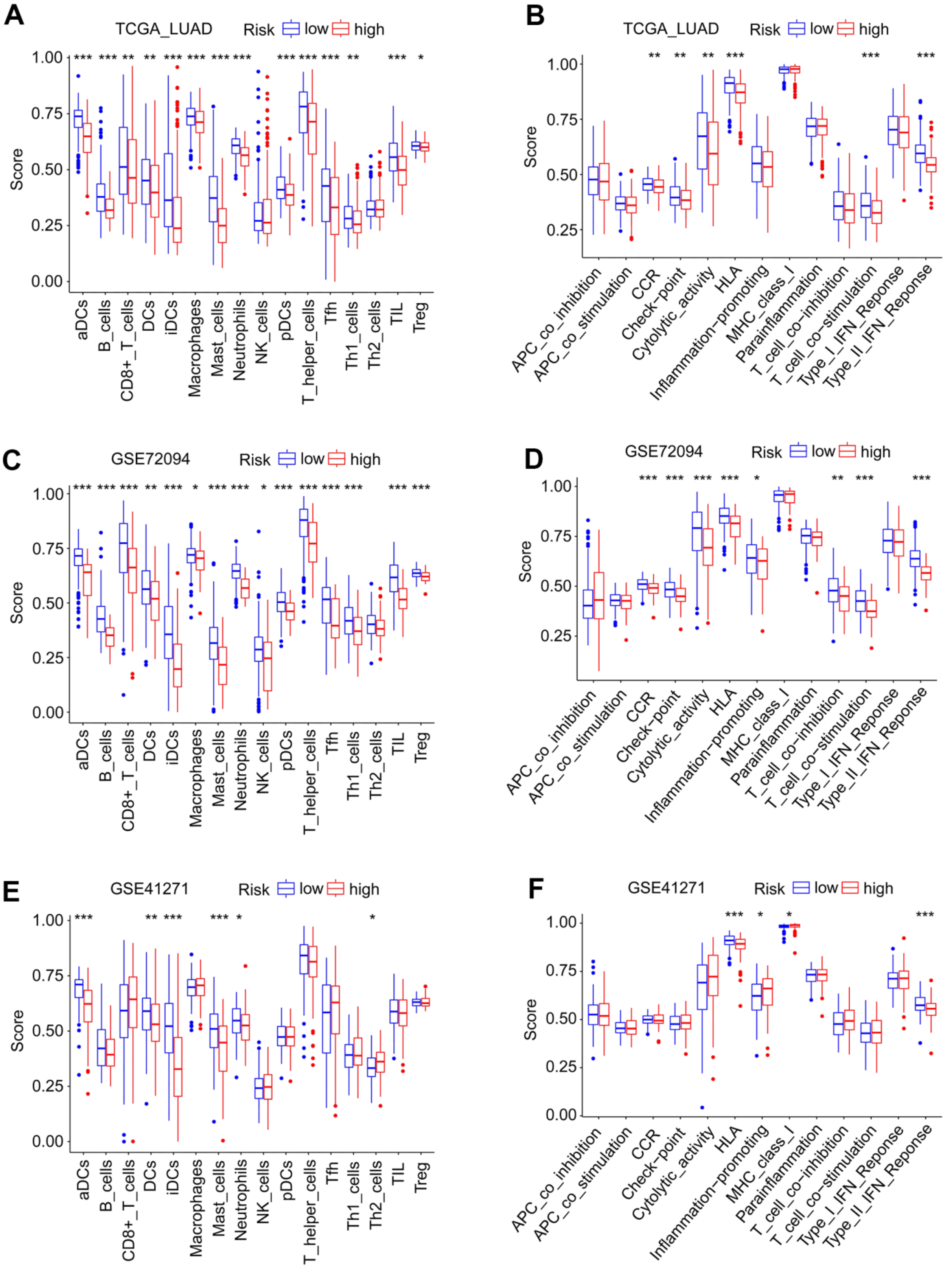

Supplementary Figure 2. Boxplot of immune cell score and immune function score based on PRRS. (**A**–**E**) Boxplot of immune cell score and immune function score based on risk score classification in TCGA_LUAD (**A**, **B**), GSE72094 (**C**, **D**), and GSE41271 (**E**, **F**) cohorts.

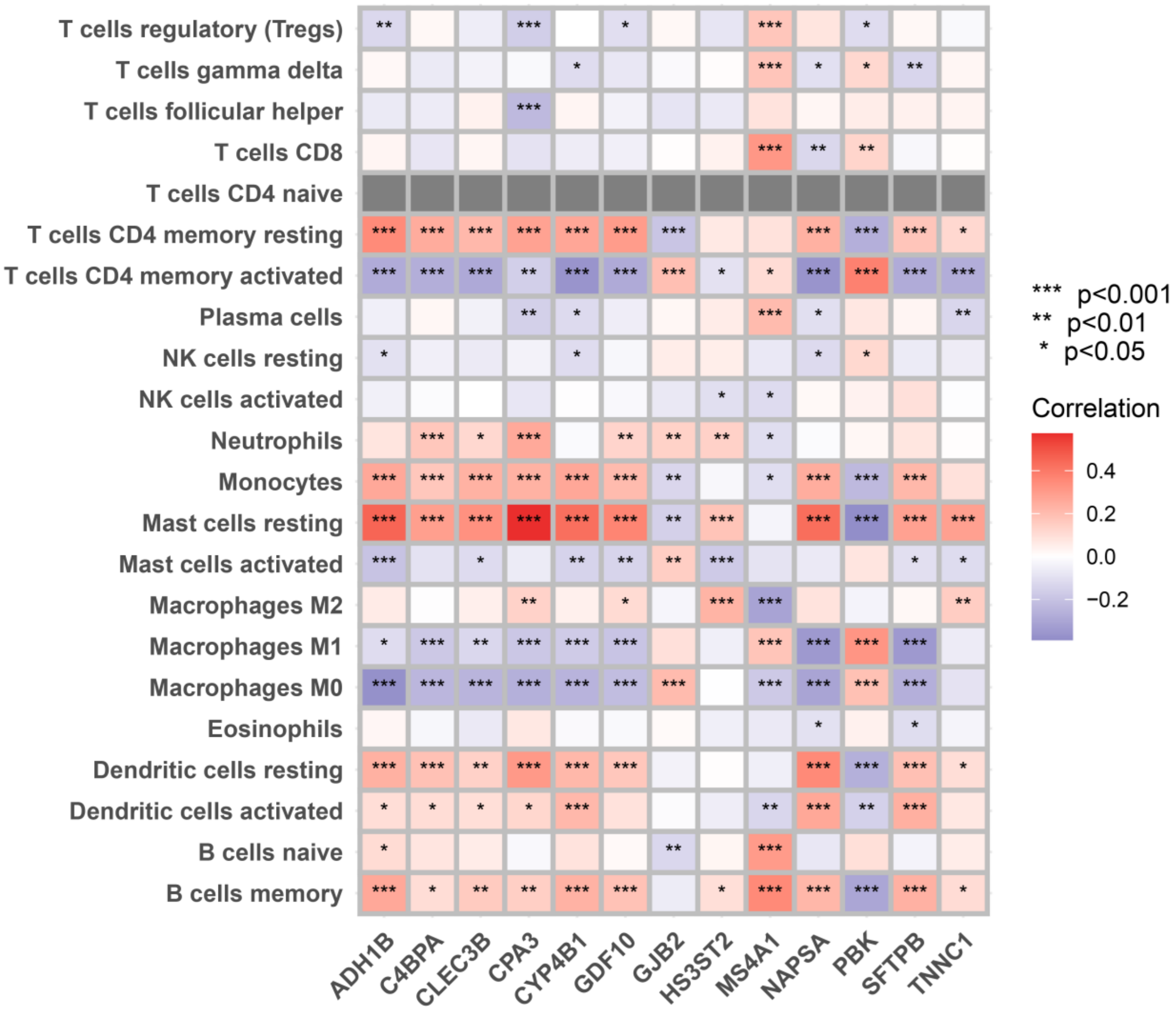

Supplementary Figure 3. Heatmap of correlation between immune cells and the 13 crucial genes.

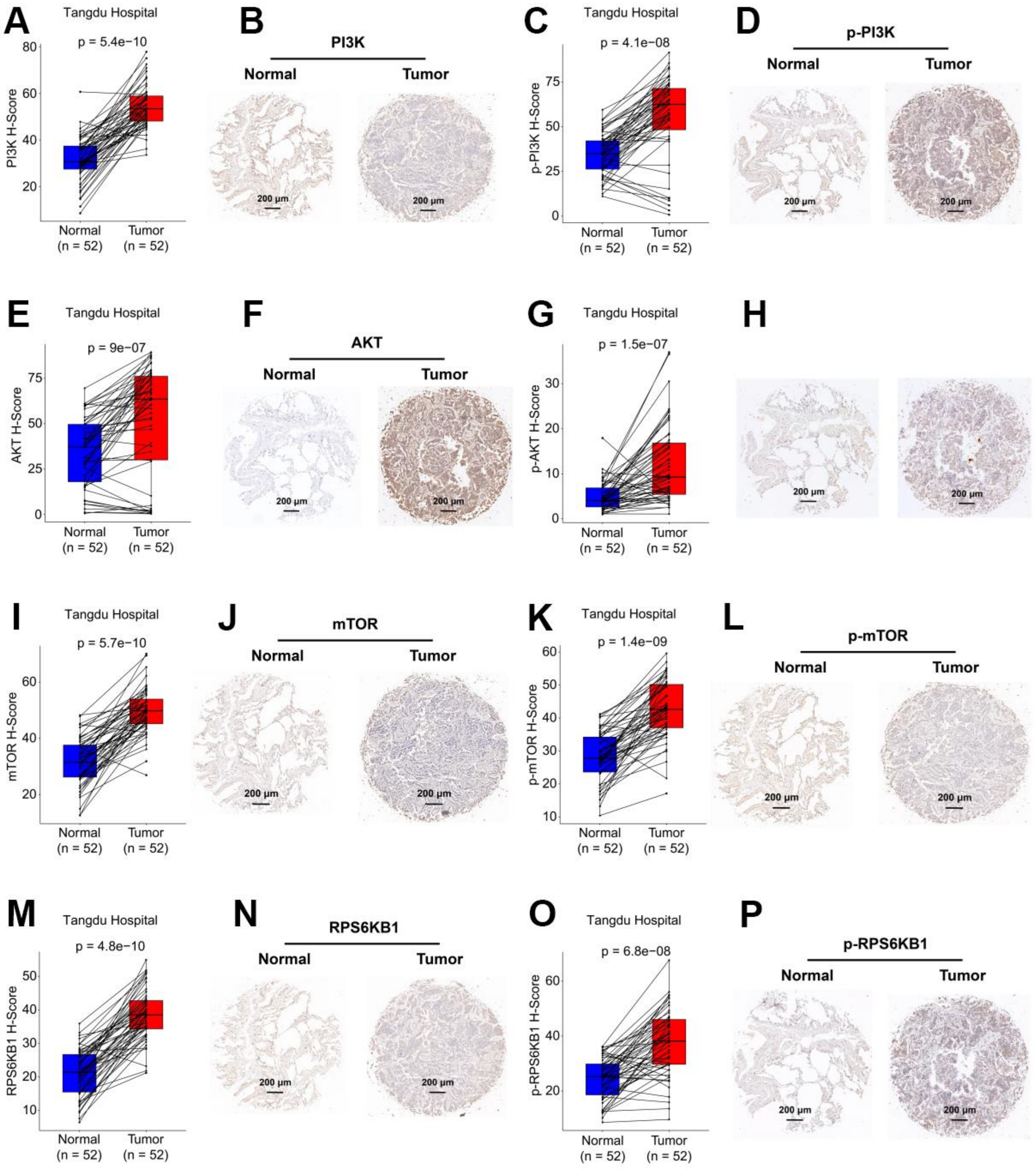

Supplementary Figure 4. Immunohistochemical detection of target gene in Tangdu Hospital cohort. (**A**–**P**) Protein levels of target genes in patients in Tangdu Hospital cohort, and representative IHC staining of indicated proteins in carcinoma and para-carcinoma tissues: PI3K (**A**, **B**), p-PI3K (**C**, **D**), AKT (**E**, **F**), p-AKT (**G**, **H**), mTOR (**I**, **J**), p-mTOR (**K**, **L**), RPS6KB1 (**M**, **N**), and p-RPS6KB1 (**O**, **P**).
