# Supplementary material for: Abnormal low expression of SFTPC promotes the proliferation of lung adenocarcinoma by enhancing PI3K/AKT/mTOR signaling transduction
Source: Aging (Albany NY). 2023 Nov 12;15(21):12451–75. doi: 10.18632/aging.205191 (PMC10683597; doi:10.18632/aging.205191)
Supplement: Supplementary Tables 1 and 2 [file aging-15-205191-s002.pdf]

SUPPLEMENTARY TABLES

Supplementary Table 1. Sequences of SFTPC-RNAi.

gene	RNAi sequence
shSFTPC#1	GCTGCTACATCATGAAGATAG
shSFTPC#2	GGTGTATGACTACCAGCAGCT

Supplementary Table 2. Antibody information.

SFTPC (DF6647, Affinity)
PI3K (AF6241, Affinity)
phospho-PI3K (AF3242, Affinity)
AKT (AF6261, Affinity)
phospho-AKT (AF0016, Affinity)
mTOR (AF6308, Affinity)
phospho-mTOR (AF3309, Affinity)
RPS6KB1 (AF6226, Affinity)
phospho-RPS6KB1 (AF3228, Affinity)
GAPDH (T0004, Affinity)
Goat Anti-Rabbit IgG (H+L) HRP (S0001, Affinity)
Goat Anti-Mouse IgG (H+L) HRP (S0002, Affinity)
